# Venues and methods to improve professional men’s access to HIV self-testing and linkage to HIV prevention or treatment: a qualitative study

**DOI:** 10.1186/s12913-021-07259-6

**Published:** 2021-11-09

**Authors:** Patience A. Muwanguzi, Esther M. Nasuuna, Florence Namimbi, Charles Peter Osingada, Tom Denis Ngabirano

**Affiliations:** 1 School of Health Sciences, College of Health Sciences, Makerere University, Kampala, Uganda; 2 Infectious Diseases Institute, College of Health Sciences, Makerere University, Kampala, Uganda

**Keywords:** Workplace HIV testing, HIV self-testing, Men, Urban, Sub-Saharan Africa

## Abstract

**Background:**

HIV testing among men in sub-Saharan Africa is sub-optimal. Despite several strategies to improve access to underserved populations, evidence regarding engaging men in professional and formal occupations in HIV testing is limited. This study explored employed professional men’s preferences for uptake of HIV self-testing, and linkage to HIV care, or prevention services.

**Methods:**

This was an explorative-descriptive qualitative study where a sample of 33 men from six Ugandan urban centres. Participants were purposively selected guided by the International Standard Classification of Occupations to participate in in-depth interviews. The data were collected using an interview guide and the sample size was determined by data saturation. Eligibility criteria included fulltime formal employment for over a year at that organization. The data were analyzed manually using thematic content analysis.

**Results:**

Three categories emerged: uptake of HIV self-tests, process of HIV self-testing and linkage to post-test services. The different modes of distribution of HIV self-test kits included secondary distribution, self-tests at typically male dominated spaces, delivery to workplaces and technology-based delivery. The process of HIV self-testing may be optimized by providing collection bins, and mHealth or mobile phone applications. Linkage to further care or prevention services may be enhanced using medical insurance providers, giving incentives and tele counselling.

**Conclusion:**

We recommend utilization of several channels for the uptake of HIV self-tests. These include distribution of test kits both to offices and men’s leisure and recreation ‘hot spots’, Additionally, female partners, peers and established men’s group including social media groups can play a role in improving the uptake of HIV self-testing. Mobile phones and digital technology can be applied in innovative ways for the return of test results and to strengthen linkage to care or prevention services. Partnership with medical insurers may be critical in engaging men in professional employment in HIV services.

## Background

HIV testing uptake among men in sub-Saharan Africa remains sub-optimal, however, linkage to care represents the one of the greatest challenges to achieving the Joint United Nations Programme on HIV/AIDS (UNAIDS) targets [1]. In 2020, knowledge of HIV status was lower among men (79%) than women (87%) across sub-Saharan Africa, but the largest gap was among men aged 35–49 years, with an estimated 701,000 remaining undiagnosed [2]. In 2019, 12% of the new infections and 28% were among men aged 15–24 and 25–49 respectively in East and Southern Africa [1]. In Uganda, approximately 62% of men living with HIV, knew their HIV status in 2019 [3], while the estimated incidence among adults aged 15–64 years in urban areas was 0.44% compared to 0.39% in rural areas, with the annual incidence peaking among men aged 35–49 years (0.47%) [4]. This is the typical peak age of employment. Therefore, reaching working men with HIV testing and treatment coverage could be critical to reducing HIV incidence among women in sub-Saharan Africa, and by extension, reducing mother-to-child transmission [2].

Men in sub-Saharan Africa report that they face stigma when they go to take an HIV test at a health facility, thus the preference for community-based HTS [5]. Stigma exists when “elements of labeling, stereotyping, separation, status loss, and discrimination occur together in a power situation that allows them” [6]. In this case, this stigma may arise from individuals, community members and health workers negative beliefs, attitudes, and fears [7]. Therefore, strategies for improving men’s uptake of HIV testing service, have focused not only on clinical-based populations but also emphasize community-based services [8, 9] such as home-based testing, workplace-based testing, and partner testing. Findings from men in rural Zambia suggest that community-based testing reduces the cost and inconvenience associated with travelling to health facilities [10]. Although community-based HIV testing may reduce stigma, it still does not overcome some of the barriers such as long waiting lines when men go to health facilities to initiate antiretroviral therapy (ART) [5]. Therefore, the provision of treatment at non-facility testing sites through differentiated service delivery may help in addressing some of these challenges [11]. A study on community influences on married men’s uptake of HIV testing in Chad, Ghana, Malawi, Nigeria, Tanzania, Uganda, Zambia, and Zimbabwe recommended flexible hours, expanding mobile clinics, and private access to care are also recommended [12, 13]. Campaigns to increase the uptake of HIV testing using cash and non-monetary incentives have reported varying levels of success [14]. In south Africa, a mobile clinic that combined testing and incentives reported a positivity yield (15%), however, this was among youth in informal dwellings with high unemployment rates which may raise ethical concerns such as potential for coercion [15].

HIV self-testing (HIVST) is another strategy that has shown success in reaching high-risk men and first-time testers [16]. According to the World Health Organization (WHO), this strategy reduces men’s interaction with the healthcare system for testing, overcomes several masculinity issues and can be distributed in various creative ways [17]. Despite the high uptake of HIV self-testing in non-facility-based testing, linkage to HIV confirmatory testing, ART initiation and prevention services remained a challenge in Malawi and Zambia and other sub-Saharan countries [18–22]. WHO recommends self-testing at the workplace as a potential strategy to reach men, since studies report one of the key reasons for their lack of HIV testing and care as their busy work schedule [23]. The International Labour Organization (ILO) classifies occupations into ten major groups, Managers, Professionals, Technicians and associate professionals, Clerical support workers, Service and sales workers, Skilled agricultural, forestry and fishery workers, Craft and related trades workers, Plant and machine operators, and assemblers, Elementary occupations and Armed forces occupations [24]. A few studies assessing HIVST in workplaces in sub-Saharan Africa have been conducted at mining and farming industries (Malawi, Zambia, and Zimbabwe) [16], at truck yards (Kenya) [25], farms, agricultural industries, factories, construction sites and taxi ranks (South Africa) [26]. These studies have largely been conducted among men in informal types of employment. We did not find published literature regarding HIV testing and linkage practices targeting men employed in professional and formal occupations, Managers, Professionals, Technicians and associate professionals, Clerical support workers, and Service and sales workers. The men in these types of occupations typically work regular hours which are normally the same as clinic opening hours and may therefore not engage in HIV services due to their work schedules. They are also at higher socio-economic status; higher education level and national HIV policy makers may assume that they have adequate knowledge regarding HIV, and thus may not be prioritised while designing national HIV testing plans. This may omit an unidentified transmission source of the HIV infection, since some of these men may be living with HIV but unaware of their status. Furthermore, there is limited information generally regarding what works for reaching men, and there is need to reach ‘older men’ over 35 years old for HIV services [27]. Given that there is a paucity of literature regarding this population, we set out to explore professional men’s preferences for uptake of HIV self-testing and linkage to post-test services in Uganda.

The study was conducted during the COVID-19 pandemic in Uganda, and data were collected between September and November 2020.

## Methods

### Research team and reflexivity

The research team members are all health professionals. PAM, EMN, TDN and CPO have received extensive training in qualitative research methodology, while EMN, and FN work in the field of infectious diseases, specifically HIV. The research assistants have all undergone training in qualitative research methods and re-trained at the start of this project. PAM kept a daily self-reflective journal before and during the study period. This journal included the researcher’s assumptions, beliefs, opinions, and an analysis of how these might affect the study process and study outcomes.

### Study design and participants

This was an explorative-descriptive qualitative (EDQ) study among men in six urban Ugandan towns and cities. The EDQ design was chosen because of its suitability for studies aiming to explore preferences, concerns, attitudes, and perception [28]. The study was conducted in the following urban sites: Kampala, Gulu, Hoima, Wakiso, Mbale and Mbarara. A list of urban centres from the Uganda Bureau of Statistics (UBOS) [29], was categorized by region. We then selected the six (6) centres from each region using simple random sampling. The men were purposively selected to participate in in-depth interviews. The eligibility criteria included: Working at a professional/ formal job, regular hours, > one year, and holding a full-time position. We selected the cut-off of one year, with the assumption that after one year in the role, one may be comfortable enough to make suggestions regarding their welfare at the workplace.

### Selection of participants

In the first instance, we contacted the office of the chief administrative officer in each town to identify the medium and large-sized workplaces. We sought administrative clearance from each individual workplace and email addresses for potential participants from the human resource officer at each work setting. We approached workplaces that had over 50 male professional employees. Participants were purposively sampled to include men from all employment levels ranging from senior management, middle management, lower-level management, supervisors, and employees. Additionally, we selected men from diverse professions, occupations, and roles, and from both public and private sectors. We identified men in different professional roles and selected one person per professional role or category. The aim was to elicit heterogeneous responses with the assumption that each man’s perceptions may also be influenced by their different professions or occupations. The selection of occupations was guided by the International Standard Classification of Occupations (ISCO) [24]. Using this approach, 122 men received an email with details of the study and as part of their consent, were requested to provide a phone number, Skype® address or receive a Zoom® meeting link depending on their preferred mode of contact. Of the 122 men who were approached, 57 declined to participate. Therefore, 65 consented and were enrolled. Data collection stopped when no new information emerged from the interviews, indicating that the study had gained maximum information about the phenomenon after 33 interviews.

### Data collection

Due to the COVID-19 restrictions and country research guidelines, we conducted one-time phone, Zoom®, or Skype® in-depth interviews (IDI). The interviews were conducted by PAM and two research assistants from 1st September to 26th November 2020, and each lasted 45 min to one hour.

Data collection employed an interview guide. The guide was developed by the research team and pilot tested among a similar population in another district. The interview guide included questions about the men’s perception of HIV testing at the workplace, HIV self-testing, ways to improve uptake of testing at the workplace and their preferences for linkage to care or prevention services following a positive or negative self-test result, respectively. The researchers listened to each interview recording daily to identify further areas for probing questions. The probing questions included modes of delivery of the HIV self-test kits and follow-up after a self-test. All the interviews were audio recorded with the participants’ permission.

### Data analysis

The data were manually analyzed using thematic content analysis [30]. As this was an EDQ study, we used the thematic content approach because there was no prior research or theoretical definition on this phenomenon to offer guidance on the themes [31]. The first author (PAM) transcribed the recordings and together with another author (TDN), immersed themselves in reading the transcripts. Then the transcripts were read again and each of the analysts noted preliminary interpretations of the text while performing open coding. Another member of the team reviewed the codes from both coders to achieve consensus. That additional team members arbitrated between the coders where there was no consensus. All the initial codes highlighted with similar colours were then clustered together into categories and sub-categories which captured the essence of the participants’ descriptions. These observations are reported under the four categories in the results section.

## Results

### Participant’s characteristics

Thirty-three men participated in-depth interviews (IDI). The ages of the participants ranged from 25 to 55 years. They had all attained tertiary/post-secondary level of education and 5/33 (15.2%) had taken an HIV test in the last two years. Table 1 presents the characteristics of the men.

**Table 1:** Partcipants characteristics

Enrolment, N	33
Age (years)	
Mean (SD)	41.6 (8.42)
Median (IQR)	42 (35–47)
Categorical, n (%)	
18–25	1 (3.0)
26–35	8 (24.2)
36–45	12 (36.4)
46–55	12 (36.4)
Occupation (ISCO)^a^	
Managers	7 (21.2)
Professionals	16 (48.5)
Technicians and associate professionals	6 (18.2)
Clerical office workers	4 (12.1)
Highest education level	
Diploma	4 (12.1)
Bachelor’s degree	6 (18.2)
Post graduate diploma	7 (21.2)
Professional certification	6 (18.2)
Master’s degree	8 (24.2)
PhD	2 (6.1)
Took HIV test < two years ago	
Yes	5 (15.2)
No	28 (84.8)
Willing to take HIVST at work	
Yes	24 (72.7)
No	9 (27.3)

^a^*ISCO* International Standard Classification of Occupations

### Men’s preferences for HIV self-testing and posttest services

**Fig. 1:**
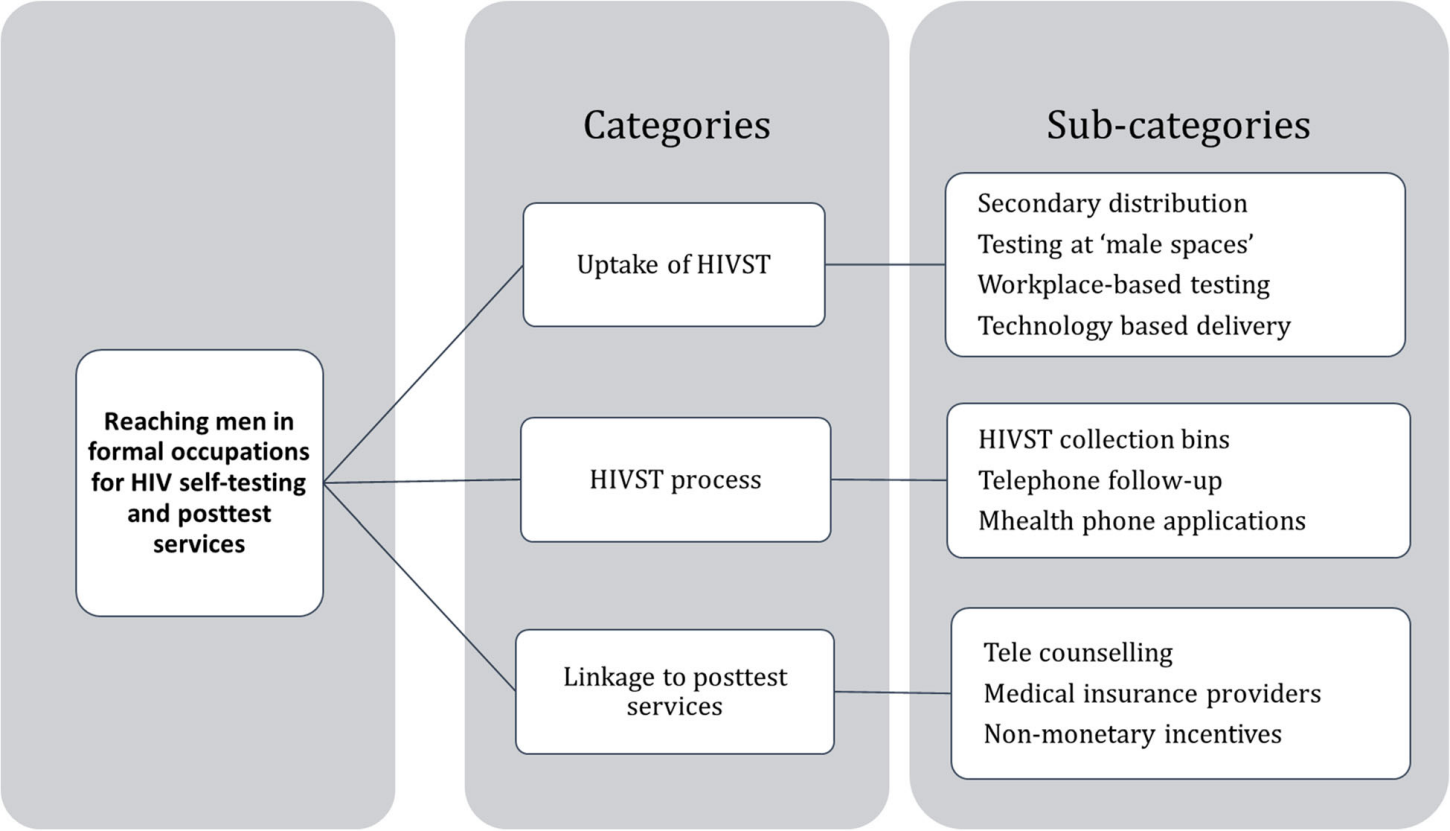
Coding tree for reaching men in formal employment for HIV self-testing and posttest services

We report the findings generated from our study in three categories: uptake of HIV self-testing, process of HIV self-testing and linkage to post-test services. The coding tree of the categories and sub-categories is presented in Fig. 1.

### Uptake of HIV self-testing

**Fig. 2:**
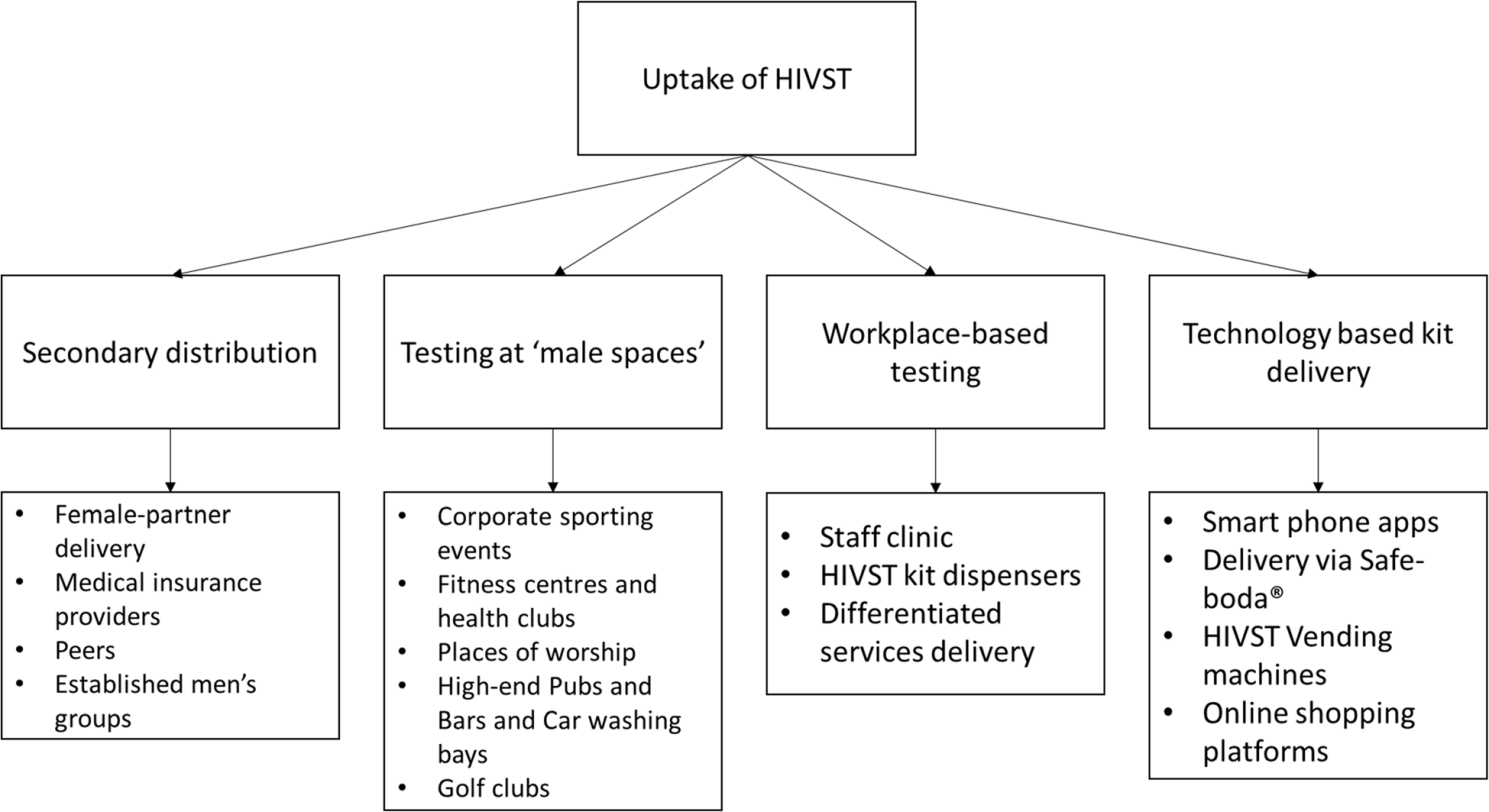
Preferences for the uptake of HIVST among men in formal employment

We asked the participants the best way to distribute HIVST kits to them. Four sub-categories emerged for the distribution channels of HIV self-test kits including secondary distribution, testing at typically ‘male spaces’, workplace delivery and technology-based delivery. The categories and sub-categories are presented in Fig. 2.

### Secondary distribution

#### Female-delivered HIVST

This involves giving the test kits to men’s female work colleagues to share with their partners. The participants presumed that men who work in professional jobs, probably had partners of similar social and economic standing, and therefore had the same characteristics such as difficulty accessing HIV testing or limited time to go to the health facility for HIV testing services. Another proposed way was to identify female groups such as church groups and through their leaders, send test kits to their partners. Therefore, they suggested the use of their female colleagues to reach other men.

*Give our female colleagues the testing kits to reach their partners at other workplaces. When they test here, they can receive a test kit for their partner and give you the partner’s phone number that way, you can reach out to the partner after maybe a week to follow-up.* (IDI, 04)

#### Peer distribution

Several participants suggested that one of the ways to get anyone involved is using peers, a particularly useful group to influence and encourage positive behavior. One participant explained:

*Men can be influenced by friends, role models and like-minded people. So, the kits can be delivered by work colleagues or champions. If this person takes up HIV self-testing, then other people are likely to take it up as well. Identify community and workplace champions, who can either create awareness or who can lead by example.* (IDI, 17)

#### Established men’s groups

Participants suggested that men have established groups such alumni groups, savings groups, and social media networking groups among others and that these groups are usually consistent with their income level or their socioeconomic status. They recommended secondary distribution of HIV self-test kits using these networks.

*I think men have groups where they belong, these maybe physical of virtual groups. For example, savings groups and alumni associations. They will typically meet regularly or have some annual meetings depending on why the groups were formed. The test kits can be distributed through the organizers or they can provide an avenue for reaching these men. Some of these schools like from all-boys schools have thousands of members.* (IDI, 14)

Another member proposed groups at churches:

*You can even distribute the test kits through the different men’s church groups or even the women’s groups to distribute to their partners.* (IDI, 06)

### Testing at ‘male spaces’

The participants highlighted the existence of ‘male spaces’, which are places dominated by men of a certain status or places where men typically meet after hours or at the weekend. The recommended venues include fitness centres and health clubs, places of worship, pubs and bars, car washing bays, golf clubs and corporate sporting events.

#### Corporate sporting events

Most participants mentioned sports events such as the corporate league or the annual marathons, although currently this might be difficult with the COVID-19 restrictions. One shared:

*Corporate men usually patronize corporate sports events because these are the ways in which they can market their company products, meet with other people and network. If you talk to the organizers, you could set up your tent or send the information beforehand that there will be HIV self-testing.* (IDI, 08)

#### Fitness centres and health clubs

More than a few participants suggested that men who work in offices could be targeted at high-end fitness centres, saunas, and gymnasiums (gyms). One participant expounded:

*Many men who work in offices, spend the greatest part of the day at their desks. Therefore, they usually like to go to the gym for a workout either in the evening or early in the morning, or at the weekend. I suggest that you go to gyms that have relationships and packages with corporate companies, high-end ones, gyms in residential areas and those at 3- and 4-star hotels.* (IDI, 07)

#### Places of worship

Some men who work in offices go to places of worship on Friday, Saturday or Sunday depending on their religion. Some participants suggested this as a potential way for reaching men.

*You can speak to the religious leaders because some of these men really trust them. The leaders can use their platforms to raise awareness for HIV testing and then on a particular day of worship, you can distribute the test kits.* (IDI, 12)

#### Pubs and bars

Participants recommended pubs and bars as potential places to get men to take an HIV test. They however expressed concern about taking the test while they are inebriated and the possibility of personal or social harm if they received a positive test result.

*There are certain types of pubs and bars that are typically frequented by corporate men, where they go to relax and unwind after a long day or week. I see it as a potential place where one can reach men with the services. However, it might be complicated with someone taking the test under the influence of alcohol, you never know what they may do when they find an unexpected result.* (IDI, 24).

#### Car washing bays

Many individuals take their cars for a wash either on Saturday evening or on Sunday evening in Ugandan towns. These bays have now expanded to provide drinks and barbecue services, and become a place where friends meet, more so in the COVID-19 era. Some participants proposed that this would be a good place to reach men for HIV testing.

*The washing bay would be a good place; you can usually find men there in small groups. Therefore, if one member of the group takes the HIV test kit, then it is likely that the others will at least consider taking one as well. Therefore, if you look for up-market car washing bays with proper seating especially close to residential areas, you will find men.* (IDI, 02)

#### Golf clubs

Some participants proposed the golf clubs as potential places to reach men of middle and higher incomes for HIV testing.

*The men that go to a golf club are those people with big salaries like CEOs. Talk to the chairperson of the golf association or just meet the management and then you can provide testing services when they have tournaments or during the weekend when most people play.* (IDI, 30)

### Workplace-based testing

#### Staff clinic

Some places of work have a staff clinic for their employees and the participants proposed this clinic as a venue for HIV self-testing.

*I think if you go to the staff clinic where we go for our treatment like malaria, you can work with the health workers there and men can pick the kits. It will also help when I have any questions, I can talk to the health workers. However, I will not give them my results, those ones I will take to the hospital.* (IDI, 09)

#### HIVST kit dispensers

A few participants suggested that the same way they receive condoms in the washrooms, a separate dispenser could be put for the HIV self-test kit. One man shared:

*A few years ago, one of the HIV projects installed condom dispensers in the male washrooms. Why can’t you also put that for the test kits because it is private? I can pick the kit and take the test from the washroom and dispose of the kit immediately.* (IDI, 18).

#### Office health and wellness events

Almost every participant reported that they have annual or regular office health and wellness events. They proposed this as a quick way to reach men for HIV self-testing. One shared:

*Every year, we have these wellness events. They usually have tests for eyes, dental services, blood pressure and talk to us about circumcision and prostate cancer screening. There is also HIV testing and blood transfusion services, however, people rarely go to the tent for HIV testing. I think the self-testing is a good way because you pick up your kit and test in private, in addition to the other health services.*

(IDI, 18)

### Technology-based HIVST kit delivery

#### Smart phone applications

Several participants suggested providing a special number to use for social media communication, and to place orders for their test-kit delivery. However, they needed to be reassured about the confidentiality of their information. One professional shared:

*If you provide a number which has Facebook® and WhatsApp® and such similar platforms, when I'm ready I can then send a message to place my order, and someone can either call me back or just work on the order.* (IDI, 20)

Another participant suggested using existing transportation phone applications. He proposed:

*These days, I use the SafeBoda® app to get items delivered fast and securely since they use motorcycles. I think if you worked with them, you could add a feature where men just placed an order for a self-test kit on the SafeBoda® app, and get it delivered to the client anywhere within their operational radius.* (IDI, 04)

#### HIVST digital vending machines

Some of the men proposed accessing the test kits from a specially designed digital vending machine. One participant expounded as follows:

*You can make a vending machine that contains several sexual and reproductive health commodities like condoms, lubricants, and the self-testing kits. One can enter a phone number to access the kit, then check the records later to see which phone number picked up a kit.* (IDI, 03)

#### Online shopping platforms

Due to the COVID-19 pandemic and global progress, many individuals including those in Low- and Middle-Income Countries are shopping online. Some of the men proposed that HIV self-testing could leverage on such platforms to distribute the kits to men who work in offices. This is an excerpt from an interview:

*The kit can be included on the platform as one of the items for sell, and the people who are doing the HIV self-testing can be the sellers. For example, an online shopping platform like Jumia with a large market share in Uganda is a good place to start. Jumia offers door-to-door delivery services, so that way the kits reach the user and will be delivered all you have to do is call the client for follow-up.* (IDI, 27)

### HIVST process

#### HIVST collection bins

One participant suggested that we can provide a bin or a locked storage container in the washroom, where participants can put their used test kits after reading the test result. He suggested the installation of locked collection sanitary bins in the washrooms such that men could place the test kits there immediately after a test, and these would be picked up regularly to confirm the results. This meant that each participant’s test kit would be clearly linked to either a phone number or a participant identifier number. This would then allow those that have not deposited their kits to be identified and followed up by a phone call. He shared:

*I think you can put a locked bin in the washrooms and people can place their test kits there after testing. Someone can check regularly and empty the bin and then see our results. I am not sure for how long the results remain valid though.* (IDI, 10)

#### Telephone follow up

Participants proposed telephone follow-up as one of the easiest ways to get people to return the test results. They recommend that this should be discussed beforehand so that a participant is given the choice for how they would like the test results to be returned. One participant elucidated:

*All men in office jobs have mobile phones and they go everywhere with them. If you discussed beforehand that someone should be called after a certain period to return the test results, when you call, he will respond. Do not underestimate the power of telephone calls because that way the patient or health worker can also ask any follow-up questions that they have.* (IDI, 27)

#### Mhealth phone applications

The men put forward that smart phone applications such as Facebook or WhatsApp could be a potential channel for the testers to return their results. They also proposed the development of specific apps to encourage HIV testing. One participant expressed as follows:

*I think for me the easiest way would be send a real time picture of the test result and then dispose of the kit immediately. Almost all of us use WhatsApp or Facebook or even email, if you promise that my results will not be shared anywhere. A new application for this kind of thing could also be developed, where for example the phone can read and upload test results* (IDI, 17)

Another participant proposed below:

*… smart phones can be an extremely useful tool, for the sake of confidentiality, we can just send a text message with a code that either says “yes or no” of “1 or 2” such that whoever reads that message will not know what we are talking about. However, the code should be sufficient for the message recipient to know whether the sender has tested positive or negative.* (IDI, 23).

### Linkage to confirmatory testing, ART initiation and prevention services

#### Tele counselling

The men proposed that they could receive telephone counselling to answer questions and for the health workers to follow up for further testing. Furthermore, the participants suggested that the person who will conduct virtual posttest follow-up should meet the men before they take the test, this will establish rapport and instil confidence when the men must divulge their test results.

One shared:

*I think the phone can be used for follow-up and support after getting one’s test results. In this era of COVID and with our busy schedules it's very hard for me to keep going to the facility, but if I have somebody to continually offer guidance and information, I think it makes it easier.* (IDI, 18)

#### Medical insurance providers

Several of the men recommended liaising with their medical insurance service providers because they have databases of all the beneficiaries receiving medical coverage. They advised that these databases could be used for contacting the men for the initial uptake of HIVST, and further engagement after the test. One participant shared:

*We all receive health insurance, therefore, if you get in touch with our insurance providers and you discussed at that level, in conjunction with the human resource officer then you can get information about the men. You can access phone numbers you or email addresses and contact them regarding the testing. It is also important that you connect with the health facilities providing care such that if one tests positive, it becomes quite easy to get treatment as part of the medical insurance plan.* (IDI, 25).

#### Non-monetary incentives

Several of the participants reported that they needed some form of motivation or incentive to take this test. They suggested that the incentives should be non- monetary and gave several examples of incentives that would motivate them to take the test. One participant shared:

*Why would I want to take an HIV test? There must be a reason why, especially if you want me to take it at the office. But if you put a test and said those who take the test and go for treatment will be given something, then I would be willing to participate.* (IDI, 11)

Another participant expounded:

*No, I do not want money but maybe mobile data, or a gym membership discount, supermarket vouchers or maybe discounts for my monthly TV subscription.* (IDI, 29).

Several participants suggested that the incentives could also be used to enhance linkage to further services.

*You could even motivate them that you will give them a reward when they bring back the results or when they go for other services. For example, circumcision or when they start on treatment if they test positive.* (IDI, 33).

## Discussion

The results of this explorative-descriptive qualitative study provide insights into professional men’s preferences for accessing HIV self-testing (HIVST) services in Uganda. We focused on men working in professional and formal occupations, who were employed in full-time positions and had worked for a year at the same organization. Although these men are highly educated with 100% attaining a post-secondary qualification, only 15.2% had taken an HIV test in the last two years.

Three categories emerged from the interviews: uptake of HIVST, Process of HIVST and linkage to post-test services. The men proposed different modes of distribution of kits such as secondary distribution of test kits, self-testing at typically male dominated spaces, delivery to workplaces and technology based the delivery of the self-test kit. They suggested that test results could be returned using collection bins and giving incentives and tele counselling to enhance linkage to further care and services. This agrees with findings from a study by Parish and colleagues among dental professionals in USA where they recommended greater support from national dental organizations, and dental insurance companies to enhance uptake of HIV testing in dental settings [32].

Our findings indicate that men are amenable to female-delivered HIV self-test kits and they propose various ways. Female partner delivered HIVST has been used successfully among pregnant women in during antenatal care [33–35] and female sex workers [36, 37]. Some studies have reported challenges with this method including lack of immediate counselling, concerns around trust, and intimate partner violence (IPV) or verbal abuse depending on how the woman presented the matter or if the man was taken unawares [38]. A study in Uganda did not report any serious adverse events among individuals that self-tested for HIV following female-delivered HIVST, while women in Malawi reported verbal or physical abuse and economic hardship [39]. Another difficulty with this method is that it relies on the solidity of the couple’s relationship. It can be affected by the gendered power dynamics and control over decision-making in the household, for instance if the woman does not usually have a lot of say in the home [40]. On the other hand, this strategy encourages HIV-status disclosure and adherence to HIV treatment in future if they test positive, because they will test together as a couple. Matovu et al. suggest some strategies to optimize the female delivered HIVST approach including maintaining open communication throughout the process, placing kits in conspicuous locations in the house, and seeking support from health workers [41].

Increasing the number of distribution channels of HIV self-test kits in key ‘hot spots’ of men is vital to reaching them for HIV testing [42]. One recurrent discovery in this study was finding the men where they are. This aligns well with the Ugandan MoH guidelines which recommend novel methods to engage men in HTS including testing at workplaces, and key populations hotspots [43]. Some studies have been conducted around finding men at such settings including venues for televised football matches in Uganda [44], workplaces [16, 25], community centres, at male-dominated workplaces and at venues including sporting events, tuckshops, bottle shops and other public gatherings in South Africa [26]. We therefore propose the identification of ‘male spaces’ in communities and working with community leaders and champions to provide creative dissemination strategies for HIV self-testing that are user friendly and convenient.

Technology based delivery was another important distribution method for the study participants. This includes the use of interventions that have been reported with some success such as smart phone applications [45, 46], HIVST electronic vending machines [47, 48] and online shopping platforms [49], although there are challenges with such unsupervised and unregulated distribution models. The World Health Organization (WHO) recommends that appropriate instructions, information and support, as well as contact details should be provided with the kit when HIVST kits are distributed directly to the user [50].

The participants recommended the use of collection bins for returning test kits. This method reduces the likelihood of accidental disclosure and solves the challenge of where to safely dispose the used test kit [51]. Although this method may be feasible as part of a research study, it may not be easy to scale up due to the number of workplaces nationwide but could be considered for places with a large workforce. Additionally, it may place health workers at additional risk as they would be required to sift through medical waste.

Telephone follow up and tele counselling emerged repeatedly from our findings as the men’s preferred method to support their linkage to confirmatory testing, ART initiation and prevention services. All the study participants possessed mobile phones, which is not surprising in a country with about 25 million estimated cellular phone subscribers [52]. Mobile phones could be used to send reminders and health education information. It should be noted that they requested a discussion prior to HIVST as they were worried that somebody else would access their phone and cause unintentional disclosure. Furthermore, the men expressed the need for confidentiality and as such proposed the use of platforms that have a facility for video or face-to-face calls. Telehealth approaches of delivering HIV testing and care are currently being trialled in some settings [53, 54], while telephone follow-up has been successful in several studies [26, 55–57]. However, some of the challenges included some men giving wrong numbers, others refusing to pick their calls, while other phones went to voicemail. We therefore recommend the use of telehealth models to improve men’s engagement at different points of the HIV care continuum.

One strategic finding that arose was the recommendation to liaise with medical insurance providers. Several studies on HIV self-testing have listed the cost as one of the barriers to the uptake of testing. Estem et al. suggest that offering HIV self-tests through medical insurance programs may be an effective approach and overcome the challenge of the cost, as this would be covered as part of the insurance scheme [42]. The insurance providers would also play a pivotal role in the initial sensitization and creation of awareness about testing and follow up after testing.

### Strengths and limitations

The strength of this study is that it is the first to exclusively document the preferences for HIV self-testing among professional men. The findings provide insight into how this underserved population can be reached to participate in these initiatives and importantly this knowledge lays the foundation for strategies to for linkage to treatment and care in the event of reactive self-test results. As this was a qualitative study, participants were purposively selected from different work settings. By design qualitative studies do not result in generalizable findings but rather our results may be transferable to similar settings. To improve transferability, we endeavoured to select participants from different ranks, professions, and sectors. Additionally, we involved participants from six Ugandan districts.

On the other hand, the interviews that were conducted by phone made it impossible to observe for non-verbal cues, therefore, in places with good internet connectivity, we prioritized the use of Skype® and Zoom® video calls.

## Conclusions

This study explored the professional men’s preferences for access to HIV self-testing, and linkage to care, or prevention services and revealed that some men were not aware of their HIV status. This study suggests several strategies to optimize the uptake of HIV self-testing among men in formal employment, however further quantitative and experimental research is needed to evaluate the effectiveness of these strategies in Uganda and in other sub-Saharan African contexts. Additionally, linkage to care remains a challenge and further research to determine the strategy with the highest proportion of men linked to HIV treatment and care following positive self-test results is warranted.

We make the following recommendations based on our study findings. First, we suggest the utilization of various channels to distribution HIV self-test kits including workplace-based testing, testing at men’s leisure and recreation ‘hot spots’, and secondary distribution through female partners, peers and established men’s groups including social media groups. Second, make the most of mobile phones to provide HIVST education and innovative ways for returning test results and to encourage linkage to post-test services and technology such as online shopping platforms and vending machines. Third, we recommend strengthening partnerships with medical insurers since they have access to both the employees and the health facilities. Finally, we recommend working with key stakeholders including community leaders, religious leaders, employers, leaders of established men’s groups and health professionals as champions to increase the uptake of HIV testing and enhance linkage to post-test services.

## Data Availability

All relevant data have been presented in this paper and the raw data is available from the authors upon reasonable request.
